# Role of Gaming Devices Associated With Internet Gaming Disorder in China: Cross-sectional Study

**DOI:** 10.2196/40130

**Published:** 2023-01-16

**Authors:** Yifan Li, Ying Tang, Shucai Huang, Linxiang Tan, Qiuping Huang, Xinxin Chen, Shuhong Lin, Jingyue Hao, Zhenjiang Liao, Hongxian Shen

**Affiliations:** 1 Department of Psychiatry National Clinical Research Center for Mental Disorders The Second Xiangya Hospital of Central South University Changsha China; 2 Department of Psychiatry The Fourth People’s Hospital of Wuhu Wuhu China; 3 Education Center for Mental Health Central South University Changsha China; 4 Department of Applied Psychology School of Humanities and Management Hunan University of Chinese Medicine Changsha China

**Keywords:** internet gaming disorder, gaming device, gaming motivation, personality traits, gaming use characteristics

## Abstract

**Background:**

Chinese gamers use computer and mobile phone games widely. Consequently, concerns regarding the development of internet gaming disorder (IGD) in adolescents have been raised. However, only a few studies have focused on the influence of gaming devices on IGD.

**Objective:**

This study aims to compare sociodemographic information, gaming use characteristics, personality traits, and gaming motivations between computer game users (CGUs) and mobile phone game users (MGUs), as well as identifying IGD predictors.

**Methods:**

This was a cross-sectional study. A total of 3593 internet game players took part in an online survey, which included sociodemographic information, gaming patterns, gaming motivations, the Chinese version of the Video Game Dependency Scale, and the Chinese Big Five Personality Inventory brief version. The population was divided into 2 groups for comparison by mobile phone or computer use, and the IGD population was also compared within the 2 groups.

**Results:**

There were significant differences between the 2 gaming device groups in the time (*t*_2994_=7.75, *P*<.001) and money (*t*_2994_=5.11, *P*<.001) spent on gaming and in internet game addiction scores (*t*_2994_=3.68, *P*<.001). Individuals using different gaming devices had different game motivations and personality traits and preferred different genres of games. Results showed that IGD predictors were different for the 2 groups, for example, strategy (odds ratio [OR] 4.452, 95% CI 1.938-10.227; *P*<.001) and action shooter (OR 3.725, 95% CI 1.465-9.474; *P*=.01) games increased the risk for MGUs.

**Conclusions:**

Gaming devices should be considered during early identification, such as long daily gaming time, much money spent on gaming, neuroticism, and conscientiousness. In addition, more research should be conducted on new gaming devices and IGD treatment.

## Introduction

Over the past few decades, internet games have been firmly integrated into people’s daily routines, especially among children and adolescents. According to the China Internet Network Information Center (CNNIC) latest report, by the end of December 2021, the number of internet users in China had reached 1.032 billion, and over 517.93 million netizens play games, accounting for 52.4% of the total number of internet users [1]. It is considered that video game activities improve basic attentional functions [2]. However, its inappropriate use has been considered a serious public health issue. Playing games for long periods can increase the incidence of dry-eye disease, obesity, and mental health problems [3,4]. Previous studies have found a positive association between digital device use and poor sleep quality and psychological distress, such as depression or internet gaming disorder (IGD) [5-8].

The American Psychiatric Association [9] first included IGD in the *Diagnostic and Statistical Manual of Mental Disorders, Fifth Edition* (DSM-5) in 2013 as a disease that needs further exploration, and in 2019, the World Health Organization (WHO) officially included it as a psychiatric disorder in the *International Classification of Diseases 11th Revision* (ICD-11) [10]. The reported prevalence of IGD varies due to differences in the measurement and sampling methods. Thus, the worldwide prevalence of gaming disorder is 3.05% [11]. In China, the prevalence of problematic gaming among adolescents is 5%-17% [6]. Furthermore, many studies have investigated the risk factors for IGD, but few studies have considered the influence of gaming devices; today, a large proportion of gamers use mobile phones, which allows them to play games limitlessly, which may increase the IGD risk.

Steam, the largest digital platform for gaming on a personal computer (PC), announced that 20,313,451 users registered on it during the coronavirus pandemic [12]. The same year, according to Google, mobile phone gamers across 10 countries increased their duration and frequency of gaming by two-thirds [13]. According to a 2020 survey, 62.5% of underage internet users in China regularly play online games. Among them, 28.9% play computer games and 56.4% play mobile phone games [14]. Owing to advances in technology, people’s entertainment has been gradually replaced by computers and video games, and many scenarios that would not happen in the real world are implemented in the virtual world, making digital games more attractive to people [15]. Games have become an increasingly popular pastime, especially during the coronavirus pandemic [16]. In today’s developed visual media, in addition to computers, mobile phones are another popular terminal for internet games. Different devices can meet different needs [17]. Computer games often feature high-quality sound and visual effects and usually require more time to play; therefore, they have the potential to provide immersion, fulfillment, and competitiveness. In addition, mobile phone games are not only easier to access than computer games but they also increase feelings of social connection and reduce feelings of isolation and negative emotions. One study found that texting games are easily associated with social networking service applications [18]. In addition, recent advances in smartphone gaming platforms have made it easier to access a wide variety of game genres [17]. Furthermore, recently, the number of mobile phone game users (MGUs) has increased dramatically, and the mobile phone gaming market has flourished, leading to a gradual shift in the traditional gaming model from desktop PC– to mobile device–based gaming models [19]. Therefore, this study aims to explore the differences between computer game users (CGUs) and MGUs to gain a deeper understanding of IGD.

In addition, exploring internet gaming use characteristics is important to better understand IGD. Internet gaming characteristics can be used as IGD predictors, including weekday/weekend game time and money spent on games, which can provide information for clinical diagnosis and treatment [20,21]. The influence of the game genre on IGD is worth mentioning. Analysis of variance reveals that internet gaming addiction varies according to the game genre [22]. A noteworthy finding was that IGD might be more prevalent in users of specific genres, especially real-time strategy and first-person shooter games [23]. Gaming motivation is another important factor for predicting IGD. Escapism motivation has demonstrated a significantly strong association with IGD [24]. Other types of motivation may also be used to predict disease [25]. Thus, the theory, assessment, and treatment of IGD can be improved by examining the association between specific types of motivation [26].

Previous studies have indicated that personality traits may also play a necessary role in the development of IGD [27]. Personality traits are characterized by high stability, reflecting thoughts, attitudes, emotions, and behavioral patterns. Despite some controversy, the 5-factor model has become a widely accepted framework of personality traits, which includes 5 dimensions: conscientiousness, extraversion, agreeableness, neuroticism, and openness [28]. Personality traits can affect an individual’s experience of games, causing players to show different degrees of pathological game use disorder [29]. Gamers with IGD show high levels of neuroticism [30]. Individuals high in neuroticism tended to experience negative emotions. The act of playing games, especially in immersive environments, provides gamers with alternative virtual reality to relieve negative moods [5]. In addition, gamers with low conscientiousness have a higher risk of developing IGD because they are less persistent in pursuing goals and focusing on responsibilities in their daily lives [31]. Individuals with IGD tend to overplay games, which can affect their relationships, work, or education. Conscientious people are less likely to allow important aspects of their lives to be negatively affected by games [32]. However, results for other personality traits have been inconsistent.

Although previous studies have reported features associated with IGD, most have focused on computer games. Given that each gaming device has unique interface characteristics, we hypothesize that there are some differences in the psychometric properties and development of IGD between CGUs and MGUs. This study aims to compare internet gaming use characteristics and personality traits between different gaming devices and to explore potential specific predictors for IGD according to different gaming devices (mobile phone or computer).

## Methods

### Study Design and Subjects

This was a cross-sectional study that collected the internet addiction scores, game time, money, game type, game motivation, and personality traits of different gaming device users through an online survey. Differences in these indicators across users of gaming devices or gaming addicts were compared to infer the characteristics of these different populations playing games and the association between devices and gaming addiction.

This study was conducted between October and November 2019 in China. We issued online questionnaires through WeChat (a cross-platform instant messaging tool), based on a network survey. For better understanding, we used the Chinese version of the scale and the Chinese questionnaire. Participants could fill in the questionnaire anonymously by scanning the quick response (QR) code of the survey program Questionnaire Star (a free online questionnaire, survey, and voting tool). On the first page of the questionnaire, we briefly introduced the purpose of this study and some matters that needed attention when filling in the questionnaire. We also demonstrated the voluntary nature, anonymity, and confidentiality of the survey. Individuals could choose the “accept” or “reject” options. Participants could choose “reject” to exit the survey page, and those who chose “accept” would skip to the next page and continue to complete the questionnaire.

The participants were recruited online through WeChat, Weibo, or other social apps. In this study, the inclusion criteria were (1) aged 15-25 years, (2) played online games in the past 12 months, and (3) understood the questionnaire content and agreed to participate. The survey initially recruited 4012 participants. Those who had never played online games or only played offline games were excluded (n=103, 2.6%). Individuals who had severe physical or mental disorders (n=87, 2.2%) were also excluded. To be specific, the following individuals were excluded: (1) current or previous history of psychiatric disorders, including but not limited to schizophrenia, bipolar disorder, or depression, and (2) current or history of medical disorder, including but not limited to neurological and endocrinological disorders. Diagnoses of schizophrenia, affective disorders, and other disorders were obtained by experienced clinicians through the DSM-5 interview [9]. For example, if a subject presented with cognitive, affective, and behavioral disturbances and severe dissonance in clear consciousness (eg, paradoxical thinking or commentary hallucinations), schizophrenia was likely to be diagnosed; if a subject presented with significant and persistent high or low mood, depressive disorder or bipolar disorder was likely to be diagnosed. Other psychiatric disorders were also diagnosed according to the criteria in the DSM-5. In addition, we recommended that these excluded individuals seek professional medical advice. Questionnaires that lacked authenticity were deleted (eg, multiple replies from the same IP address; n=55, 1.4%). In addition, we removed responses in which the gaming device was neither a computer nor a mobile phone (eg, Nintendo Switch, Sony PlayStation, or virtual reality headsets) and individuals who played computer and mobile phone games evenly (n=174, 4.3%). Finally, a total of 3593 (89.6%) internet game users were included.

### Ethical Considerations

The study protocol was approved by the Institutional Review Board. All participants were fully informed about the purpose of this investigation and signed an online informed consent form. The study was conducted in accordance with the Declaration of Helsinki, and it was approved by the Research Ethics Committee of Second Xiangya Hospital of Central South University (protocol code 2020004, date March 1, 2020).

### Procedures and Measures

Self-administered questionnaires were used to collect sociodemographic information, gaming use characteristics, IGD, and personality traits. Before starting the study, the questionnaire was tested with a small sample of adolescents to improve readability and intelligibility, and it was finalized after the preliminary study.

Sociodemographic data included gender, age, education level, marital status (married or unmarried), and structure of the family (either a sibling or an only child).

In terms of gaming use characteristics, participants were asked which gaming device they used most often (mobile phone, computer, or others).Three groups were divided by reports from the responders based on the gaming time spent on either a computer or a mobile phone: (1) computer game: individuals who played only computer games or played more computer games than mobile phone games; (2) mobile phone game: individuals who played only mobile phone games or played more mobile phone games than computer games; (3) others: individuals who played computer and mobile phone games evenly or the gaming device was neither a computer nor a mobile phone. Participants were also asked about their motivation for playing games, the game genre (role-playing games [RPGs], strategy games, action shooter games, and brain and skill games), and the time (hours/day) and money (yuan/month) spent on gaming in the past 12 months. Specifically, gaming motivation included sensation seeking, escaping reality, coping with negative emotions, passing time, and making friends.

IGD was assessed using the Chinese version of the Video Game Dependency Scale (VGD-S). The scale is a revision of the English version developed by Rehbein et al [33,34] and covers the description of IGD in the DSM-5, published by the American Psychiatric Association (Cronbach α=.92). The scale developed by Rehbein et al [34] is a 4-point Likert-type scale (strongly disagree to strongly agree) consisting of 18 items, with 2 items for each criterion. Participants were asked to choose an experience during the past 12 months from the list of 18 descriptive items. At least 1 of the 2 items must be answered with “strongly agree” to meet the diagnostic criterion. Participants who met 5 or more of the 9 criteria were placed in the IGD group (Cronbach α=.90).

We used the brief version of the Chinese Big Five Personality Inventory to measure personality traits [35-37]. The scale consists of 8 items for each subtype, and there are 40 items in total. In addition, it is scored on a 6-point Likert scale ranging from 1 (strongly disagree) to 6 (strongly agree) and demonstrates high construct validity and reliability (Cronbach α=.85).

### Statistical Analysis

First, to reduce bias and confounding variables, we used propensity score matching (PSM) to match CGUs and MGUs based on demographic information. Chi-square tests and *t*-tests(two tails) were used to compare demographic information, IGD scores, internet gaming use characteristics, and personality traits between CGUs and MGUs. Logistic regression analysis was performed to identify IGD predictors in different game device groups. A 2-sided statistical significance level was set at *P*<.05. SPSS Statistics version 26.0 (IBM Corp) was used in this study.

## Results

### Sociodemographic Data

A total of 3593 subjects were included in the analysis. Of these participants, 1511 (42.1%) were CGUs and 2032 (57.9%) were MGUs. Considering the huge number of differences, PSM was used to match the age, gender, education, marital status, and family structure (only child in the family or not). Next, we acquired 1:1-matched samples, and 1498 (99.1%) CGUs and 1498 (73.7%) MGUs were included. The mean age for each group was 19-20 years (CGU: mean 19.6, SD 1.7 years; MGU: mean 19.6, SD 1.7 years), and most of them were male (CGU: 1259/1498, 84.0%; MGU: 1270/1498, 84.8%). Almost all subjects were unmarried (CGU: 1447/1498, 96.6%; MGU: 1456/1498, 97.2%), and over half of them were an only child (CGU: 886/1498, 59.1%; MGU: 839/1498, 56.0%), which did not present a significant difference. The results of the *χ*^2^ test indicated a significant difference regarding the education level (*χ*^2^_1_=38.3, *P*<.001) of the 2 device-specific groups: the percentage of people who earned a bachelor’s degree or higher was greater in the CGU group (886/1498, 59.1%) than in the MGU group (717/1498, 47.9%); see Table 1.

**Table 1:** Sociodemographic data of the 2 different gaming device groups.

Characteristics		MGUs^a^ (n=1498)	CGUs^b^ (n=1498)
Age (years), mean (SD); *t* (*df*)/*χ*2 (*df*)=0.16 (2994), *P*=.87		19.58 (1.67)	19.59 (1.70)
**Gender, n (%); *t*(*df*)/*χ*2 (*df*)=0.31 (1), *P*=.58**			
	Male	1270 (84.8)	1259 (84.0)
	Female	228 (15.2)	239 (16.0)
**Marital status, n (%); *t*(*df*)/*χ*2 (*df*)=0.90 (1), *P*=.34**			
	Unmarried	1456 (97.2)	1447 (96.6)
	Married	42 (2.8)	51 (3.4)
**Education, n (%); *t*(*df*)/*χ*2 (*df*)=38.32 (1), *P*<.001**			
	Lower than undergraduate	781 (52.1)	612 (40.9)
	Undergraduate or higher	717 (47.9)	886 (59.1)
**Only child, n (%); *t*(*df*)/*χ*2 (*df*)=1.14 (1), *P*=.28**			
	Yes	839 (56.0)	868 (57.9)
	No	659 (44.0)	630 (42.1)

^a^MGU: mobile phone game user.

^b^CGU: computer game user.

### Comparisons of Gaming Pattern IGD Score and Game Genre Between CGUs and MGUs

Participants with IGD and the overall population were compared separately between the 2 groups (Table 2). For the overall population in the CGU group, the mean IGD score was 2.95 (SD 2.67) and the mean time and money spent were 2.21 (SD 1.56) hours/day and 111.31 (SD 189.80) yuan/month (mean US $15.95, SD US $27.20), respectively, while in the MGU group, the mean IGD score was 2.60 (SD 2.57) and the mean time and money spent were 1.95 (SD 1.26) hours/day and 63.00 (SD 149.05) yuan/month (mean US $9.03, SD US$21.36), respectively. IGD scores (*t*_2994_=3.68, *P*<.001), time (*t*_2994_=7.75, *P*<.001), and money (*t*_2994_=5.11, *P*<.001) in the MGU group were all significantly lower than those in the CGU group. The 2 most popular game genres in the CGU group were strategy (1048/1498, 70.0%) and action shooter (205/1498, 13.7%), whereas in the MGU group, the 2 most popular game genres were strategy (981/1498, 65.5%) and RPGs (200/1498, 13.4%). For subjects with IGD in the CGU group, the mean IGD score was 6.86 (SD 1.48) and the mean time and money spent were 3.08 (SD 2.00) hours/day and 228.34 (SD 238.15) yuan/month (mean US $32.73, SD US $34.13), respectively. In the MGU group, the mean IGD score was 6.74 (SD 1.46) and the mean time and money spent were 2.75 (SD 1.63) hours/day and 160.78 (SD 261.41) yuan/month (mean US $23.04, SD US $37.47), respectively. The time (*t*_681_=2.37, *P*=.02) and money (*t*_681_=3.53, *P*<.001) spent in the MGU group were significantly lower than those in the CGU group. In addition, the 2 most popular game genres in both groups were strategy (CGU: 246/369, 6.7%; MGU: 222/314, 70.7%) and RPGs (CGU: 69/369, 18.7%; MGU: 47/314 15.0%).

**Table 2:** Within-group comparisons of IGD^a^ scores, gaming patterns, personality traits, gaming motivations, and game genres.

Characteristics		MGUs^b^		CGUs^c^		Total statistics		IGD statistics	
		Total (n=1498)	IGD (n=314)	Total (n=1498)	IGD (n=369)	*t* (*df*)/*χ*2 (*df*)	*P* value	*t* (*df*)/*χ*2 (*df*)	*P* value
IGD score, mean (SD)		2.60 (2.57)	6.74 (1.46)	2.95 (2.67)	6.86 (1.48)	3.68 (2994)	<.001	1.02 (681)	.31
**Gaming patterns, mean (SD)**									
	Gaming time	1.95 (1.26)	2.75 (1.63)	2.21 (1.56)	3.08 (2.00)	7.75 (2994)	<.001	2.33 (681)	.02
	Money spent gaming (yuan/US $)	63.00 (149.05)/9.03 (21.36)	160.78 (261.41)/23.04 (37.47)	111.31 (189.80)/15.95 (27.20)	228.34 (238.15)/32.73 (34.13)	5.11 (2994)	<.001	3.53 (681)	<.001
**Personality traits, mean (SD)**									
	Neuroticism	25.43 (8.07)	29.68 (6.94)	25.22 (8.10)	29.18 (6.25)	–0.70 (2994)	.48	–0.98 (681)	.33
	Conscientiousness	33.07 (6.33)	30.65 (5.26)	33.29 (6.57)	30.42 (5.65)	0.91 (2994)	.36	–0.55 (681)	.59
	Agreeableness	34.79 (5.98)	33.02 (5.59)	34.41 (6.56)	31.80 (5.86)	–1.64 (2994)	.10	–2.76 (681)	.01
	Extraversion	29.65 (7.04)	28.53 (6.28)	30.02 (7.49)	28.91 (6.94)	1.40 (2994)	.16	0.76 (681)	.48
	Openness	32.96 (7.14)	31.90 (6.37)	34.01 (7.24)	32.45 (6.38)	3.98 (2994)	<.001	1.12 (681)	.26
**Gaming motivations, n (%)**									
	Sensation seeking	517 (34.5)	177 (56.4)	620 (41.4)	211 (57.2)	15.04 (1)	<.001	0.05 (1)	.83
	Escaping reality	163 (10.9)	86 (27.4)	221 (13.8)	14 (3.8)	10.05 (1)	.002	9.83 (1)	.002
	Coping with negative emotions	673 (44.9)	181 (57.6)	711 (47.5)	212 (57.5)	1.94 (1)	.16	<0.01 (1)	.96
	Passing time	1008 (67.3)	193 (61.5)	854 (57.0)	204 (55.3)	33.65 (1)	<.001	2.66 (1)	.10
	Making friends	298 (19.9)	73 (23.2)	384 (25.6)	107 (29.0)	14.04 (1)	<.001	2.89 (1)	.09
**Gaming genres, n (%)**									
	RPGs^d^	200 (13.4)	47 (15.0)	179 (11.9)	69 (18.7)	1.33 (1)	.25	1.68 (1)	.20
	Strategy	981 (65.5)	222 (70.7)	1048 (70.0)	246 (66.7)	6.85 (1)	.01	1.28 (1)	.26
	Brian and skill	149 (9.9)	8 (2.5)	66 (4.4)	7 (1.8)	34.52 (1)	<.001	0.33 (1)	.56
	Action shooter	168 (11.2)	37 (11.8)	205 (13.7)	47 (12.7)	4.19 (1)	.04	0.14 (1)	.70

^a^IGD: internet gaming disorder.

^b^MGU: mobile phone game user.

^c^CGU: computer game user.

^d^RPG: role-playing game.

### Comparisons of Personality Traits and Gaming Motivations Between CGUs and MGUs

Table 2 outlines the significant differences between device-specific groups regarding gaming motivations and personality traits. For the overall population in both groups, CGU participants presented higher openness than MGU participants (*t*_2994_=3.98, *P*<.001), and for subjects with IGD, CGU participants presented lower agreeableness than MGU participants (*t*_681_=−2.76, *P*=.006). Referring to gaming motivations, including sensation seeking, escaping reality, coping with negative emotions, passing time, and making friends, the two most important game motivations were passing time (CGU: 854/1498, 57.0%; MGU:1008/1498, 67.3%) and coping with negative emotions (CGU: 711/1498, 47.5%; MGU: 673/1498, 44.9%) in the two groups among the overall population. For the subjects with IGD, the most two important motivations in the CGU group were coping with negative emotions (212/369, 57.5%) and seeking sensations (211/369, 57.2%), while in the MGU group, those were passing time (193/314, 61.5%) and coping with negative emotions (181/314, 57.6%).

### Identifying IGD Predictors by Game Device Group

After controlling for sociodemographic factors, multivariate logistic regression was conducted to identify IGD predictors in the different game device groups (see Table 3). In the CGU group, a higher daily gaming time (odds ratio [OR] 1.301, 95% CI 1.182-1.432; *P*<.001), more money spent monthly on gaming (OR 1.002, 95% CI 1.001-1.003; *P*<.001), higher neuroticism (OR 1.060, 95% CI 1.038-1.083; *P*<.001), and lower conscientiousness (OR 0.922, 95% CI 0.894-0.951; *P*<.001) were associated with IGD, in addition to motivation for sensation seeking (OR 2.387, 95% CI 1.781-3.200; *P*<.001), escaping reality (OR 3.407, 95% CI 2.347-4.945; *P*<.001), and coping with negative emotions (OR 1.456, 95% CI 1.085-1.955; *P*=.01). In the MGU group, a higher daily gaming time (OR 1.377, 95% CI 1.226-1.547; *P*<.001), more money spent monthly on gaming (OR 1.004, 95% CI 1.003-1.006; *P*<.001), higher neuroticism (OR 1.087, 95% CI 1.063-1.112; *P*<.001), and lower conscientiousness (OR 0.928, 95% CI 0.898-0.960; *P*<.001) were associated with IGD, in addition to motivation for sensation seeking (OR 2.244, 95% CI 1.651-3.050; *P*<.001), escaping reality (OR 2.271, 95% CI 1.496-3.447; *P*<.001), and coping with negative emotions (OR 1.493, 95% CI 1.099-2.029; *P*=.01). Compared to brain and skill games, strategy (OR 4.452, 95% CI 1.938-10.227; *P*<.001) and action shooter games (OR 3.725, 95% CI 1.465-9.474; *P*=.01) increased the risk for the occurrence of IGD in this group, which was different from the CGU group.

**Table 3:** After adjusting for sociodemographic data^a^, binary logistic regression analyses of factors predicting IGD^b^ in device-specific groups.

Characteristics		CGUs^c^		MGUs^d^	
		OR^e^ (95% CI)	*P* value	OR (95% CI)	*P* value
**Gaming patterns**					
	Gaming time	1.301 (1.182-1.432)	<.001	1.377 (1.226-1.547)	<.001
	Money spent	1.002 (1.001-1.003)	<.001	1.004 (1.003-1.006)	<.001
**Gaming motivations**					
	Sensation seeking	2.387 (1.781-3.200)	<.001	2.244 (1.651-3.050)	<.001
	Escaping reality	3.407 (2.347-4.945)	<.001	2.271 (1.496-3.447)	<.001
	Coping with negative emotions	1.456 (1.085-1.955)	.01	1.493 (1.099-2.029)	.01
	Passing time	1.113 (0.832-1.490)	.47	0.906 (0.656-1.251)	.55
	Making friends	1.026 (0.742-1.419)	.88	0.891 (0.614-1.294)	.54
**Personality traits**					
	Neuroticism	1.060 (1.038-1.083)	<.001	1.087 (1.063-1.112)	<.001
	Conscientiousness	0.922 (0.894-0.951)	<.001	0.928 (0.898-0.960)	<.001
	Openness	0.983 (0.958-1.009)	.19	1.003 (0.971-1.031)	.98
	Agreeableness	0.990 (0.910-1.021)	.53	0.991 (0.959-1.025)	.60
	Extraversion	1.003 (0.980-1.027)	.78	1.001 (0.973-1.031)	.92
**Game genres**					
	Brain and skill	N/A^f^	N/A	N/A	N/A
	Strategy	1.486 (0.612-3.607)	.38	4.452 (1.938-10.227)	<.001
	Action shooter	1.500 (0.577-3.901)	.40	3.725 (1.465-9.474)	.01
	RPGs^g^	1.557 (0.583-4.159)	.38	2.434 (0.963-6.150)	.06

^a^Sociodemographic data included gender, age, education, marital status, and family structure (only child or note).

^b^IGD: internet gaming disorder.

^c^CGU: computer game user.

^d^MGU: mobile phone game user.

^e^OR=odds ratio.

^f^N/A: not applicable.

^g^RPG: role-playing game.

## Discussion

### Principal Findings

To the best of our knowledge, this is the first study to compare internet gaming usage characteristics and personality traits among people using different gaming devices. The main research findings are as follows: First, the game time, money spent, and IGD scores in the CGU group were higher than those in the MGU group. Second, subjects in different groups preferred different game genres and had different game motivations and personality traits; for example, subjects in the CGU group preformed lower openness than those in the MGU group. Third, the time and money spent on games, neuroticism, conscientiousness, motivation to seek sensations, escaping reality, and coping with negative feelings were associated with the occurrence and development of IGD in both groups in multivariate logistic regression; for MGUs, comparing the brain and skill, strategy, and action shooter games may bring more risks for IGD.

Several researchers have reported that time and money are dominant factors for IGD, and our study found that these 2 factors take effect in each group [20,33,38,39]. In this way, it seems that some developers of highly profitable games become whipping boys. However, in fact, most developers are just enthusiasts who want to create the most enjoyable gaming experience for their players, while game publishers are more concerned about monetization and drive developers in this way, due to which the game comes with paid content to attract players to invest as much time and money in the game as possible. Moreover, to maximize profits, the classical gaming operation pattern usually does not have fixed endpoints, which can easily cause gaming addiction [40]. The playing time, game currency input, and addiction value of the CGU group were higher than those of the MGU group. Some possible explanations are that mobile phones are currently more adapted to some medium-quality or online games with social attributes, while some computer games have higher quality, more immersive experiences, and higher prices, and high-quality games cause greater addiction problems. However, furthermore, as technology advances, the technological differences between mobile phones or other mobile devices and computers will become increasingly smaller. We can assume that mobile phone game addiction may replace computer game addiction as a larger social health issue within a few years because mobile devices are more accessible and often free to play.

The game genre plays an important role in the development of IGD, and it is important to explore its impact on IGD [41]. Our study showed that the 2 groups had different gaming preferences. For the addicted population, both groups preferred strategy and adventure games, and for MGUs, the strategy and action shooter games may bring more risks. Previous research has found that strategy games and action shooter games are popular among teenagers with high IGD risks [42]. Action shooter games have been reported to be associated with high impulsivity, disinhibition, and inattention [42]. Therefore, teenagers’ liking strategies or action shooter games need the necessary acquaintances and encouragement to engage in healthy behaviors.

Although many studies have reported the relationship between RPGs and addiction, this study did not find meaningful differences, possibly due to the heterogeneity of the subjects. The reason for the different game preferences between the 2 groups may be that the number of popular games varies on different devices, particularly when the Chinese government tightens restrictions on game distribution, which requires further research.

Certain personality traits can make individuals more prone to addiction. This study found that neuroticism and conscientiousness were both identifying factors for IGD in each group, which is consistent with previous findings [33,43,44]. Highly neurotic individuals are more likely to feel stimuli from the external environment. Online gaming is often one of the choices to gain a sense of security [31]. In addition, it is difficult for those with low levels of conscientiousness to assume appropriate responsibilities or obligations, and the online world offers an environment of less oversight and responsibility, which is tempting for them [33]. Because the 2 personality traits are significantly associated with several addiction disorders, parents and educators should not only value highly neurotic and conscientious adolescents but also help them overcome personality disorders [45,46].

We also found that the openness of the MGU group was higher than that of the CGU group. This means that people in the MGU group preferred to maintain their own gaming habits and did not easily change their game types and modes. In the IGD subgroup, the agreeableness of the CGU group was lower than that of the MGU group. Agreeableness is an assessment of the degree to which an individual likes to appear with others, and it reflects their attitude toward others. Low agreeableness can display a hostile, cynical, and ruthless attitude [47]. The finding indicates that computer game players prefer to enjoy the game alone and may encounter more social problems.

Motivations to seek sensations, escape reality, and cope with negative feelings were seen as factors strongly related to game addiction in our research, which is consistent with previous research findings [24]. In the total population, 34.5% of MGU subjects and 41.4% of CGU subjects played games because of sensation seeking, while in the IGD population, the number of people was >50%. People get pleasure from completing objectives, and developers have designed many large and small objectives in their games (eg, beating an opponent or just checking in online). Some of these in-game objectives are easy to complete, while others seem hardcore (especially those in competitive online games that require expert skills, coping, and long-term patience). These different difficulty levels allow all types of people to find their own goals in the game and earn pleasure by completing them. Some research has found that dealing with negative emotions, such as fear of failure, by imitating game characters against the real world under performance can be seen as a negative reinforcement for gaming disorder [48].

The CGU group was more motivated to look for sensations, possibly because computer games are more immersive. Players interact more with the digital world and have more fun after completing their objectives. There were >40% MGUs and CGUs whose gaming motivation was discomfort, similar to seeking sensations, among the IGD population; the 2 groups also exceeded 50%. As mentioned before, the online world offers an environment with less oversight and accountability, where players can vent their emotions unscrupulously and take less responsibility; in addition, it is noteworthy that negative emotions are often associated with high neuroticism and low conscientiousness. More than half of the participants played games to pass the time; however, interestingly, passing time did not become an IGD predictor, suggesting that most players use games in a healthy way and as a form of entertainment, and only a minority will develop IGD.

In addition, some users play games to make friends or escape reality. Some games, such as RPGs, provide a complete set of worldview settings in which people can temporarily escape from reality and meet the psychological needs of making friends [49]. This study found that people in the CGU group had a stronger motivation to make friends than those in the MGU group, reflecting that computer game players are more enthusiastic about making friends. Some studies have found that fixed social needs aggravate the progression of internet addiction [50,51]. Therefore, for computer game players, they should not only be encouraged to engage in more offline social interactions to meet their needs but also be helped in social skills.

### Limitations

This study had several limitations. First, because this was a cross-sectional study with a nonrepresentative sample, causality and generalizability were limited. Therefore, longitudinal research and recruiting more representative samples are necessary. Second, the research data were self-reported and came from an online survey, which may be biased in terms of the reliability and applicability of the conclusion; thus, comments about the game usage of participants’ relatives or acquaintances and offline assessment of IGD by clinicians can be included to obtain more accurate and realistic results in subsequent research. Third, considering the restrictions on game introduction in the Chinese Mainland, the study did not include people using new devices, such as PlayStation, Switch, or virtual reality headsets, and research on new devices can be designed for further study. Fourth, currently, there is no internationally recognized scale for evaluating game addiction; therefore, the results of this study should be based on preliminary results. Fifth, the study did not consider confounding factors, such as smoking, alcohol consumption, household income, and social status, which play important roles in gaming addiction and motivation. These data can be obtained in follow-up studies.

### Conclusion

In summary, our study showed that there are significant differences between CGUs and MGUs in gaming usage patterns, game genres, motivations, personality traits, and internet game addiction scores. Furthermore, we found that time and money spent on games, neuroticism, conscientiousness, motivation to seek sensations, escaping reality, and coping with negative feelings are associated with the occurrence and development of IGD. Given the difference between CGUs and MGUs, there are 2 important implications for clinical research on gaming addiction disorders. More attention should be paid to high-quality computer games because of their high picture quality, immersive experience, and potential adverse consequences. The other is that useful regulation for computer game players with impaired social functioning or poor personality traits ought to be implemented. However, targeted measures need to be adopted to help people with IGD, and further studies may consider longitudinal research, including new game devices, to determine the causal relationship between addiction and various devices and other factors and accompany the innovation of game devices.

## Abbreviations

CGU: computer game user
DSM-5: Diagnostic and Statistical Manual of Mental Disorders, Fifth Edition
IGD: internet gaming disorder
MGU: mobile phone game user
OR: odds ratio
PC: personal computer
PSM: propensity score matching
RPG: role-playing game

## Data Availability

The data sets generated and analyzed in this study are available from the corresponding author on reasonable request.
